# The Mini-Addenbrooke’s Cognitive Examination (M-ACE) as a brief cognitive screening instrument in Mild Cognitive Impairment and mild Alzheimer’s disease

**DOI:** 10.1590/1980-57642018dn12-040005

**Published:** 2018

**Authors:** Diane da Costa Miranda, Sonia Maria Dozzi Brucki, Mônica Sanches Yassuda

**Affiliations:** 1 Grupo de Neurologia Cognitiva e do Comportamento (GNCC), Departamento de Neurologia, Faculdade de Medicina, Universidade de São Paulo, São Paulo, SP, Brazil.

**Keywords:** dementia, cognition, Alzheimer’s disease, cognitive dysfunction, neuropsychological tests, Brazil, demência, cognição, doença de Alzheimer, disfunção cognitiva, testes neuropsicológicos, Brasil

## Abstract

The Mini-Addenbrooke’s Cognitive Examination (M-ACE) is a brief cognitive screening test that evaluates four main cognitive domains (orientation, memory, language and visuospatial function) with a maximum score of 30 points and administration time of five minutes.

**Objective:**

To assess the performance of healthy elderly, MCI patients and mild AD patients using the Brazilian version of the M-ACE.

**Methods:**

The test was applied to a group of 36 Mild Cognitive Impairment (MCI), 23 mild Alzheimer’s Disease (AD) and 25 cognitive healthy elderly. All participants were aged ≥60 years.

**Results:**

The M-ACE displayed high internal consistency (Cronbach alpha >0.8; 95% CI 0.7-0.8) and proved effective for differentiating the AD group from MCI and control groups, providing superior accuracy than the MMSE (the cut-off point of 20 points had the highest sensitivity and specificity – 95.6% and 90.16% respectively, with a high area under the curve – AUC=0.8; 95% CI 0.7-0.9). Performance on the M-ACE was strongly correlated with that of the MMSE and Functional Activities Questionnaire (FAQ). The M-ACE was not accurate in discriminating MCI from control subjects.

**Conclusion:**

The M-ACE is a brief screening test which provided high accuracy for diagnosing AD in this sample. The suggested cut-off point in this study was 20 points for AD.

## RESUMO

A Mini-Addenbrooke’s Cognitive Examination (M-ACE) consiste em um teste de avaliação cognitiva breve composta de cinco itens que visam avaliar quatro domínios cognitivos principais (orientação, memória, linguagem e função visoespacial) com pontuação máxima de 30 pontos e um tempo de administração de cinco minutos.

**Objetivo:**

Avaliar o desempenho de idosos cognitivamente saudáveis, com CCL e DA leve na versão brasileira da M-ACE.

**Métodos:**

O teste foi aplicado em um grupo de 23 sujeitos com DA provável leve, 36 CCL e 25 idosos cognitivamente saudáveis. Todos os participantes incluídos tinham idade ≥60 anos.

**Resultados:**

A M-ACE apresentou alta consistência interna (alfa de Cronbach >0,8; IC 95% 0,7 a 0,8) e mostrou-se capaz de diferenciar o grupo DA dos demais participantes, com uma acurácia superior ao MEEM. O ponto de corte de 20 pontos foi o de maior sensibilidade e especificidade (95,6% e 90,16% respectivamente) para diferenciar DA dos outros dois grupos, com área sob a curva considerada alta (ASC=0,8; IC 95% 0,7-0,9). A M-ACE apresentou forte correlação com MEEM e Questionário de Atividades Funcionais (QAF). M-ACE não apresentou boa acurácia para diferenciar CCL do grupo controle.

**Conclusão:**

A M-ACE pode ser considerada um teste rápido de rastreio com elevada acurácia no diagnóstico de DA nessa amostra. O ponto de corte sugerido neste estudo é de 20 pontos para DA.

Cognitive assessment is fundamental for diagnosis and follow-up of Alzheimer’s Disease (AD) and other dementias, as well as Mild Cognitive Impairment (MCI). In clinical practice, high patient caseloads and limited time available for consultations preclude the use of time-consuming tests in individuals with cognitive complaints. Hence, a screening test for early diagnosis is important in yielding information and allowing the provision of social and psychological support as well as best care and intervention.^1^^,^ 2

The Mini-Addenbrooke’s Cognitive Examination (M-ACE) is a five-item scale: time orientation, learning and recall of name and address, verbal fluency for animals and the clock drawing test (CDT), designed to assess four main cognitive domains: orientation, memory, language and visuospatial function, with a maximum score of 30 points and administration time of five minutes. The validation study of the scale revealed higher sensitivity than the Mini-Mental State Exam (MMSE) across all cut-off points.^3^

Given the importance of cognitive assessment for diagnosing AD and MCI and the paucity of brief cognitive screening instruments validated for use in Brazil, the objective of the present study was to assess the performance of healthy elderly, MCI patients and AD patients using the Brazilian version of the M-ACE.

## METHODS

### Participants

Twenty-three subjects with AD and 36 with MCI were recruited from the Neurology clinic and from the Referral Center for Cognitive Disorders of University (CEREDIC) of São Paulo. Probable AD was diagnosed based on the criteria of the National Institute on Aging and Alzheimer’s Association^4^ and classified as mild according to final score one on the Clinical Dementia Rating (CDR).^5^ The diagnosis of MCI was defined using the criteria of Petersen.^6^ The control group comprised elderly without cognitive complaints recruited from the community that scored<2 on the Functional Activities Questionnaire (FAQ)^7^ and greater than the education-adjusted median on the MMSE^8^ (illiterate=20 points; 1-4 years of education=25 points; 5-8 years=26 points; 9-11 years=28 points; >11 years=29 points),^9^ and ≥7 on the memory recall of the Brief Cognitive Screening Battery (BCSB).^10^ Only participants aged ≥60 years were eligible for inclusion.

All participants gave written consent. In the case of illiterate individuals or those with dementia, the form was signed by an accompanying family member or guardian.

The study was approved by the Research Ethics Committee of the *Hospital das Clínicas da Faculdade de Medicina da Universidade de São Paulo*.

### Brazilian version of the M-ACE

The original version of the M-ACE was adapted based on the Brazilian version of the ACE-R, previously translated and validated for the Brazilian population.^11^ However, the original M-ACE has three versions (A, B and C) for longitudinal monitoring; the only difference is in name and address of the memory item. Therefore, it was necessary to create new names and addresses for the other two versions (B and C). This was initially carried out by two specialists in cognition by replicating the same format as the original version (name and surname, street with double name, followed by number, city and state). The adapted versions were established after a pilot study, in which they were applied to 15 healthy individuals, aged >60 years with different educational levels to assess its applicability and determine the need for refinements. Version A used the names and addresses from the ACE-R, “Renato Moreira, Rua Bela Vista 73, Santarém Pará”, version B: “Antonio Siqueira, Rua Porto Alegre 53, Londrina, Paraná” and version C: “Marcelo Silveira, Rua Porto Feliz 83, Santana, Amapá”. (Supplement).

### Procedures

Individuals that met the inclusion criteria were assessed between October 2015 and November 2016. All participants were submitted to anamnesis, neurological examination and a test battery including the MMSE, BCSB, FAQ and the Brazilian version of the M-ACE (versions A, B and C).

### Statistical analysis

The Chi-square test was employed to compare the categorical variables between diagnostic groups. One-way ANOVA and Kruskal-Wallis tests were employed to compare the numerical variables. Spearman’s correlation coefficient was used to analyze the relationship between M-ACE scores and the other variables assessed. Values close to +1 indicate strong correlation among values, whereas values close to 0 show an absence of relationship among the variables.^12^ Cronbach’s alpha coefficient, an indicator of internal reliability, was used to analyze the internal consistency of the M-ACE and its three versions. Alpha values ≥0.60 indicate moderate consistency, whereas values ≥0.70 indicate high consistency.^13^ The diagnostic accuracy of the M-ACE, identification of the best cut-off scores for sensitivity/specificity and the positive/negative predictive values of each of the measures were derived using the Receiver Operating Curves (ROC curves) method. The data were analyzed using the R 3.4.3 statistics software program. The level of significance adopted for the statistical tests was 5%, corresponding to a p-value<0.05.

## RESULTS

Demographic data and performance on the main cognitive tests are summarized in Table 1. Mean age of the participants was 73±7.4 years. Overall, females predominated in the groups (67.8%). Mean education was 11±5.6 years. The AD group had worse performance on all cognitive tests compared with both the MCI and control groups. There was a difference in age and sex between the groups. Regarding education, the three groups were similar.

**Table 1:** Demographic data and performance on the cognitive tests.

	Total (n=84)	Control (n =25)	MCI (n=36)	AD (n=23)	p-value
Female, n (%)	57 (67.8)	15 (60)	30 (83.3)	12 (52.2)	0.028^a^
Age, mean (SD)	73 (7.4)	70 (6.9)	72 (6.8)	77 (7.5)	0.003^b^
Education, mean (SD)	11 (5.6)	12 (5.5)	11 (5.3)	9 (5.7)	0.078^c^
M-ACE, mean (SD)	22 (6.4)	26 (3.4)	25 (3.4)	14 (4.7)	< 0.001^c^
MMSE, mean (SD)	26 (3.3)	28 (2.1)	27 (1.7)	22 (3.6)	<0.001^c^
BCSB DR, mean (SD)	7 (2.5)	8 (0.9)	8 (1.5)	4 (2.4)	<0.001^c^
FAQ^1^	3 (5.6)	0 (0.2)	0 (0.7)	10 (6.5)	<0.001^c^

SD: Standard Deviation; MCI: Mild Cognitive Impairment; AD: Alzheimer's Disease; M-ACE: Mini-Addenbrooke's Cognitive Examination; MMSE: Mini-Mental State Exam; BCSB DR: Brief Cognitive Screening Battery - Delayed Recall; FAQ: Functional Activities Questionnaire;

1 Higher scores indicate poorer functional performance;

a Chi-square test;

b One-way ANOVA;

c Kruskal-Wallis Test.

**Figure 1:**
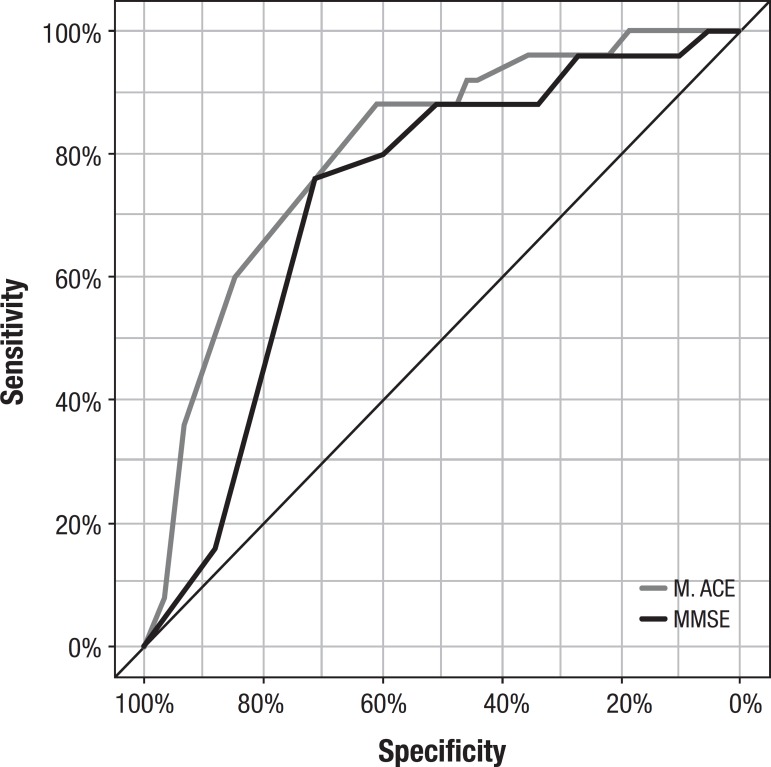
M-ACE and MMSE ROC Curves for differentiating the AD group from the other groups (MCI + Control)

The M-ACE displayed high internal consistency (Cronbach alpha=0.8, 95% CI 0.776-0.869), and high accuracy for differentiating the AD group from the other groups (MCI + Control), with greater area under the curve than the MMSE (Table 2, Figure 1).

**Table 2:** Sensitivity, specificity, PPV, NPV of the M-ACE versus MMSE (AD versus MCI and Control).

	M-ACE	MMSE
Sensitivity	95.65	91.30
Specificity	90.16	80.33
PPV	78.57	63.64
NPV	98.21	96.08
Accuracy	91.67	83.33
Cut-off point	20	26
AUC (95% CI)	0.805 (0.70-0.90)	0.726 (0.61-0.83)

Metric: Youden Index; PPV: Positive predictive value; NPV: Negative predictive value; AUC: Area under the curve; CI: Confidence interval.

**Figure 2:**
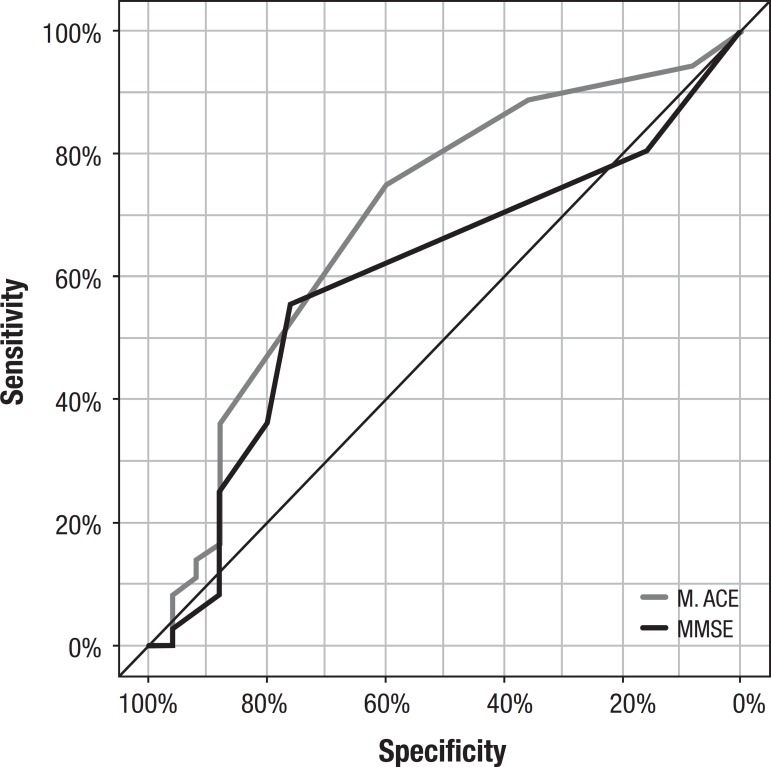
M-ACE and MMSE ROC Curves for differentiating control group from MCI group.

In terms of differentiating controls from MCI, both the M-ACE and MMSE showed low accuracy (Table 3, Figure 2). On the analysis of the confusion matrix, overall, the M-ACE had slightly better performance in discriminating the three groups compared with the MMSE (Tables 4 and 5)

**Table 3:** Sensitivity, specificity, PPV, NPV of the M-ACE versus MMSE (control versus MCI).

	M-ACE	MMSE
Sensitivity	75	55.56
Specificity	60	76
PPV	72.97	76.92
NPV	62.50	54.29
Accuracy	68.85	63.93
Cut-off point	27	28
AUC (95% CI)	0.69 (0.55-0.83)	0.60 (0.45-0.74)

Metric: Youden Index; PPV: Positive predictive value; NPV: Negative predictive value; AUC: Area under the curve; CI: Confidence interval.

**Table 4:** Confusion matrix for M-ACE.

		Predicted			
		AD	MCI	Control	Total
**Actual**	AD	**22**	1	0	23
	MCI	4	**23**	9	36
	Control	2	8	**15**	25
	Total	28	32	24	**84**

Global accuracy*=71.43%. *Obtained by dividing total correct classifications (sum of main diagonal) by total number of participants.

**Table 5:** Confusion matrix for MMSE.

		Predicted			
		AD	MCI	Control	Total
**Actual**	AD	**21**	1	1	23
	MCI	9	**11**	16	36
	Control	3	3	**19**	25
	Total	33	15	36	**84**

Global accuracy*=60,71. *Obtained by dividing total correct classifications (sum of main diagonal) by total number of participants.

On the correlation analysis, the M-ACE showed a negative correlation with age (-0.3, 95% CI=-0.5-[–0.1], p<0.001) and positive with education (0.4, 95% CI=0.2-0.5, p<0.001) and strong correlation with the MMSE (0.7, 95% CI=0.6-0.8, p<0.001) and FAQ (–0.7, 95% CI= –0.8-[–0.6], P<0.001).

## DISCUSSION

The M-ACE demonstrated easy application and good acceptability among the participants. Mean administration time of the test in the sample studied was five minutes (±2), the same as that reported for the original version.

The M-ACE proved effective for differentiating the AD group from the other groups and provided superior accuracy to the MMSE. The cut-off point of 20 points had the highest sensitivity and specificity for AD diagnosis. The M-ACE showed high reliability, indicating that all components of its score contribute to the cognitive assessment.

These results are similar to those of the validation study of the Spanish version of the M-ACE conducted in Barcelona with a sample of 175 individuals aged > 65 years. In the study, a cut-off point of 16 was defined for dementia, with sensitivity of 86.7%, specificity of 87% and AUC=0.94, and provided better discrimination rates than the MMSE (cut-off ≤24; sensitivity=88.0; specificity=78.3). The test also had high internal consistency (Cronbach alpha=0.828).^14^

**Figure 3:**
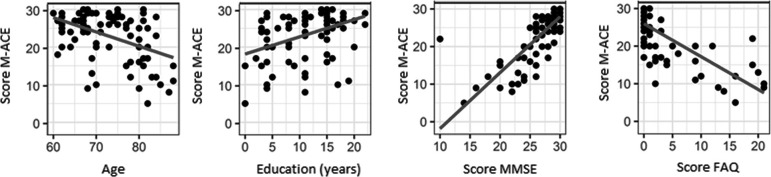
Scatter plots of M-ACE for age, education, MMSE and FAQ.

These results support the fact that, although the MMSE is the most widely used screening instrument, it has several limitations. One of these limitations is that executive function is not well assessed by the instrument, being the first (sometimes only) symptom to present in many cases of dementia syndrome. In addition, the MMSE has overly simple tasks for memory and language functions which fail to detect early deficits.^15^^,^ 16

In the M-ACE, the inclusion of the memory component (repetition and recall of name and address) reflects the importance of episodic memory impairment in early detection of AD. The M-ACE also includes the verbal fluency (animals category) test, which relies on frontal executive function and also assesses semantic memory. The incorporation of the clock drawing test (CDT) in the M-ACE further broadens its ability to analyze cognitive profile, since it assesses memory, motor function, executive function and verbal comprehension.^16^^,^ 17

In this study, the accuracy of the M-ACE in diagnosing MCI was poor. Validation studies of screening tests for MCI are scarce. A systematic review and meta-analysis pooled data in the literature on the performance of the main brief screening tests found that the MMSE had combined sensitivity and specificity of 0.62 and 0.87, respectively; the MoCA had sensitivity of 0.89 and specificity of 0.75; and the Mini-Cog sensitivity of 0.91 and specificity of 0.86. However, these results are limited because the ideal approach is to directly compare the screening tests in the same group of participants with similar educational levels.^18^

The MoCA is the most described and used test for screening MCI.^19^^,^ 20 A prospective study directly comparing the accuracy of the M-ACE with the MoCA found that both tests had good sensitivity for diagnosing dementia and MCI, a high negative predictive value, but low specificity and a positive predictive value (sensitivity of M-ACE for dementia=0.98 and MCI=0.95. Sensitivity of the MoCA for dementia=1.00 and MCI=0.92). On the analysis of the ROC curve, both showed good accuracy for dementia (AUC M-ACE=0.90 [0.87-0.93], AUC MoCA=0.91 [0.89-0.93]), where the MoCA was slightly superior to the M-ACE for detecting MCI (AUC M-ACE=0.78 [0.75-0.81], AUC MoCA=0.82 [0.79-0.85]).^21^

On the analysis of correlation of the M-ACE with demographic variables, there was positive correlation of education with total score on the M-ACE. An expected finding given that most cognitive tests are vulnerable to educational bias, an influence that is greater in countries with high levels of illiteracy and low education among elderly, such as Brazil.^22^ Analysis of the correlation of M-ACE with other cognitive tests revealed a strong correlation with the MMSE, confirming its criterion validity.

Limitations of the present study include the fact that no measurements of test stability (intra-examiner and inter-examiner) were performed. In addition, this was a preliminary assessment of the M-ACE, and application of the test in different samples is necessary.

In summary, the M-ACE is a brief screening test that provided high accuracy for diagnosing AD in this sample, but was not accurate in discriminating patients with MCI from control subjects, where more studies evaluating its performance in this group are needed.

Compared with the MMSE, the M-ACE has advantages not only regarding its psychometric properties, but also in defining cognitive profile. The test is also freely available, unlike the MMSE, whose copyright belongs to *Psychological Assessment Resources*, limiting its application, especially in research settings.

The M-ACE should be compared against other brief tests reported in the literature, such as the CASI-S and Mini-Cog, to further validate the instrument.
